# Ethnobotanical appraisal and cultural values of medicinally important wild edible vegetables of Lesser Himalayas-Pakistan

**DOI:** 10.1186/1746-4269-9-66

**Published:** 2013-09-14

**Authors:** Arshad Mehmood Abbasi, Mir Ajab Khan, Munir H Shah, Mohammad Maroof Shah, Arshad Pervez, Mushtaq Ahmad

**Affiliations:** 1 Department of Environmental Sciences, COMSATS Institute of Information Technology, Abbaottabad, Pakistan; 2 Department of Plant Sciences, Quaid-i-Azam University, Islamabad 45320, Pakistan; 3 Department of Chemistry, Quaid-i-Azam University, Islamabad 45320, Pakistan

**Keywords:** Ethno-medicinal, Cultural values, Wild edible vegetables, Lesser Himalayas

## Abstract

**Background:**

The association among food and health is momentous as consumers now demand healthy, tasty and natural functional foods. Knowledge of such food is mainly transmitted through the contribution of individuals of households. Throughout the world the traditions of using wild edible plants as food and medicine are at risk of disappearing, hence present appraisal was conducted to explore ethnomedicinal and cultural importance of wild edible vegetables used by the populace of Lesser Himalayas-Pakistan.

**Methods:**

Data was collected through informed consent semi-structured interviews, questionnaires, market survey and focus group conversation with key respondents of the study sites including 45 female, 30 children and 25 males. Cultural significance of each species was calculated based on use report.

**Results:**

A total of 45 wild edible vegetables belonging to 38 genera and 24 families were used for the treatment of various diseases and consumed. Asteraceae and Papilionoideae were found dominating families with (6 spp. each), followed by Amaranthaceae and Polygonaceae. Vegetables were cooked in water (51%) followed by diluted milk (42%) and both in water and diluted milk (7%). Leaves were among highly utilized plant parts (70%) in medicines followed by seeds (10%), roots (6%), latex (4%), bark, bulb, flowers, tubers and rhizomes (2% each). Modes of preparation fall into seven categories like paste (29%), decoction (24%), powder (14%), eaten fresh (12%), extract (10%), cooked vegetable (8%) and juice (4%). *Ficus carica* was found most cited species with in top ten vegetables followed by *Ficus palmata*, *Bauhinia variegata*, *Solanum nigrum*, *Amaranthus viridis*, *Medicago polymorpha*, *Chenopodium album*, *Cichorium intybus*, *Amaranthus hybridus* and *Vicia faba*.

**Conclusions:**

Patterns of wild edible plant usage depend mainly on socio-economic factors compare to climatic conditions or wealth of flora but during past few decades have harshly eroded due to change in the life style of the inhabitants. Use reports verified common cultural heritage and cultural worth of quoted taxa is analogous. Phytochemical analysis, antioxidant activities, pharmacological applications; skill training in farming and biotechnological techniques to improve the yield are important feature prospective regarding of wild edible vegetables.

## Introduction

Since the beginning of human civilization, people have used plants as food and medicine. The ethnobotanical pharmacology is as old as man himself. In Indo-Pak first record of plant medicine were compiled in Rig Veda between 4500–1600 BC and Ayurveda between 2500–600BC [1]. Ethnobotany deals with past and present interrelationships between human cultures and the plants. The investigation of the cultural values of plant species plays a significant role to modern medicine, farming, pharmaceutical and nutraceutical industrial sectors of a society [2]. The diversity in wild plant species contributes to household food security and health [3,4]. There are over 20,000 species of wild edible plants in the world, yet fewer than 20 species now provide 90% of our food [5]. Evidence indicates that more than 300 million people throughout the contemporary world gain part or all of their livelihood and food from forests [6].

Wild edible plants play an important socio-economic role as medicines, dyes, poisons, shelter, fibers and religious and cultural ceremonies [7]. About 46% of world’s poor live in South Asia [8] of which 75 million dwell Himalayas [9] and the biomass extraction is most widespread pressure on forests [10]. Despite agricultural societies’ primary reliance on crop plants, the tradition of eating wild plants has not completely disappeared, because of their nutritional role and health benefits. However, consumption is determined less by calorie input and more by the pleasure of gathering wild resources, recreating traditional practices and enjoying characteristic flavors [11]. Both food and medicinal plants have interventional uses. Food can be used as medicine and vice versa.

Previous epidemiologic studies have consistently shown that diet plays a crucial role in the prevention of chronic diseases [12]. This convincing evidence suggests that a change in dietary behavior such as increasing consumption of fruit, vegetables, and grains is a practical strategy for significantly reducing the incidence of chronic diseases. Consumption of vegetables, as well as grains, has been strongly associated with reduced risk of cardiovascular disease, cancer, diabetes, Alzheimer disease, cataracts, and age-related functional decline [13-16]. The relationship between food and health becomes increasingly significant as consumers now demand healthy, tasty and natural functional foods that have been grown in uncontaminated environment [17]. Knowledge of such foods is a part of traditional knowledge which is mainly transmitted through contribution of individuals of households [18]. In many parts of the world the traditions of using wild edible plants as food and medicine are at risk of disappearing, hence it is of outmost importance to obtain data about popular uses of such plants species before this knowledge disappears [19,20].

## Materials and methods

### Study site description

**Figure 1:**
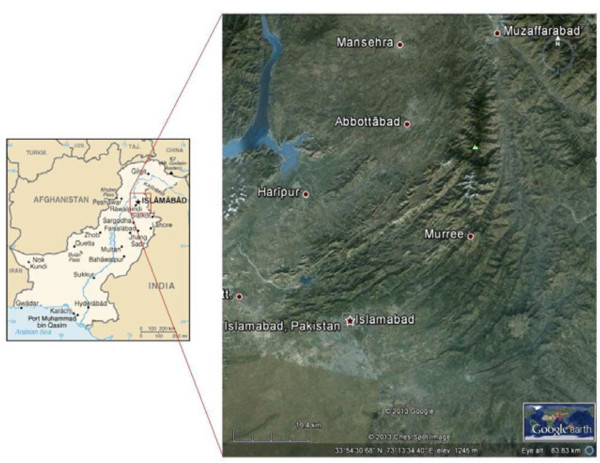
Location map of the study area.

Present study was conducted from January 2010 to May 2012 in different sites of Lesser Himalayas. Data was collected from 85 localities of five major sampling sites including Abbottabad, Haripur, Mansehra districts of Khyber Pakhtunkhwa province (KPK), Margalla Hills National Park Islamabad, Murree Hills and its allied areas in the province of Punjab (Figure 1). The Himalaya range occupies in Pakistan the regions of Kashmir, Kaghan, Kohistan, Deosai and Chilas. The Lesser Himalaya is a prominent range 2,000 to 3,000 meters elevation and lies between 33°-44′ and 35°-35′ north latitude and between 72°-33′ and 74°-05′ east longitude. The total area of Lesser Himalayas in Pakistan is proximately 23295 km^2^[21] and the total population is proximately 10 million [22]. Due to variation in the topography, altitudes aspects and vegetation cover, the climate of Lesser Himalayas ranges show tremendous variation. It falls into two major categories includes, Subtropical continental lowland including the plain and foot-hills zone and Subtropical continental highland including outer and middle Himalayas, Siwalik hills, Murree hills and entire Hazara hills. The average rainfall varies from 70–90 mm in southern parts whereas 100–130 mm in northern parts. A large part of the winter precipitation from the western disturbance is received in the form of snow. The northern parts receive little rain but heavy snowfall in the winter [21,23]. The vegetation of Lesser Himalaya falls within the subtropical, temperate, sub-alpine and alpine zones. The range management divided the area into six vegetation zones, namely; subtropical sub-humid zone, subtropical humid zone, temperate humid zone, sub alpine zone and glaciers or snow fields [21].

### Ethnobotanical study

Ethnobotanical data was collected through well-versed semi-structured interviews, questionnaires, and focus group conversation with (125) key informants ranged from 15–70 years including farmers, herdsmen, shepherds, housewives, school boys and children having sound traditional knowledge of useful wild edible plants [11,24]. Adult female members from the household, accountable for food preparation, were considered as the key respondents with supplementary information from children and adults which facilitate in collection and processing of wild edible vegetables [18] and their answers were noted verbatim [25].

Questions about wild edible vegetables consumption were mainly focused on local name of plant, part/parts of the plants used, flowering period, place, season and quantity of collection, habitat, mode of preparation and consumption, medicinal uses (method of preparation, mode of application, diseases cured), other ethnobotanical uses as (fodder, fuel, ornamental purpose, fencing, construction etc.) and threats to wild edible vegetables. Authenticity of the claims was verified by cross checking of the collected data at different villages either by showing the fresh specimen, telling local names or showing field photographs to local informants.

A total of 45 wild edible vegetables were included in the present investigation based on their use in the study area. Taxonomic identification of the collected plant samples was carried out with the help of flora of Pakistan [26]. The plant specimens were properly pressed, dried and mounted on standard size of herbarium sheets. The voucher specimens were deposited in the Herbarium of Pakistan, Department of Plant Sciences, Quaid-i-Azam University, Islamabad.

### Cultural importance index (CI)

In order to find out cultural significance of each species in every locality cultural importance index (CI) was calculated as the summation of the use report (UR) in every use category mentioned for a species in the locality divided by number of participants (N) in that locality. Similarly mean cultural importance index (mCI) of each, specie was also deliberate [11]. The cultural significance of each family (CIf) was calculated by adding cultural importance index (CI) of the species from each family [27].

$$\mathrm{CI}=\overset{i=\mathrm{NU}}{\underset{i=1}{\sum }}\frac{\mathrm{URi}}{N}$$

### Threats to wild edible vegetables

In order to know local peoples’ awareness on various activities intimidating wild edible plants, a number of threatening factors like agricultural, over grazing, harvesting and fire were identified with the community [28]. The factors were presented to informants to choose from. Then the scores from each respondent summed up, the ranks determined and the factor that received the highest total score ranked first.

## Results and discussion

### Taxonomic diversity

Flora of Lesser Himalayas is a rich source of diverse useful plant species. A total of 45 wild edible vegetables belonging to 38 genera and 24 families are collected and consumed by the local inhabitants of the study areas as mentioned in Table 1. Asteraceae and Papilionoideae were observed dominating families with (6 spp. each), followed by Amaranthaceae and Polygonaceae (4 spp. each), whereas rest of the families were represented by 2 and one species. Growth forms indicated that herbs were dominating (89%) followed by trees (9%) and shrubs (2%).

**Table 1:** Medicinal values of wild edible vegetables of lesser Himalayas-Pakistan

**S. No**	**Botanical name/Family**	**Part used**	**Mode of preparation and application**	**Diseases cured**
1	*Amaranthus hybridus* L.	Seeds	Dried seeds are grinded and powder is taken orally with water.	Eye vision
	Amaranthaceae			
2	*Amaranthus spinosus* L.	Seeds	Dried seeds powder is taken orally with water.	Eye vision
	Amaranthaceae			
3	*Amaranthus viridis* L.	Seeds	Dried seeds powder is taken orally with water.	Eye vision
	Amaranthaceae	Roots	Fresh roots are crushed and paste is applied topically	Scorpion sting
4	*Digera muricata* L.	Leaves	Fresh leaves are cooked in water and paste is taken orally.	Constipation
	Amaranthaceae			
5	*Dryopteris ramosa* (Hope) C. Chr.	Leaves	Leaves are cooked as vegetable and taken orally.	Gastric ulcer, constipation
	Aspidiaceae			
6	*Bidens bipinnata* L.	Leaves	Fresh leaves extract is applied topically.	Leprosy and skin cuts
	Asteraceae			
7	*Cichorium intybus* L.	Leaves	Fresh leaves decoction is taken orally	Fever, gas trouble, and body swelling
	Asteraceae			
8	*Launaea procumbens* (Roxb.) Ramayya & Rajagopal	Leaves	Fresh leaves are grinded along with sugar and extract is taken orally	Painful micturation
	Asteraceae			
9	*Sonchus asper* L.	Leaves	Leaves decoction is taken orally	Fever, constipation
	Asteraceae			
10	*Sonchus oleraceous* L.	Leaves	Fresh leaves decoction is taken orally	Body weakness, constipation
	Asteraceae			
11	*Taraxacum officinale* L.	Rhizome	Fresh rhizomes decoction is taken orally	Jaundice
	Asteraceae			
12	*Bombax malabaracum* (DC.) Schott & Endlicher	Bark	Fresh bark is crushed and past is applied topically	Skin eruptions, pimples, joint pain
	Bombacaceae			
13	*Capsella bursa*-*pastoris* (L.).	Leaves	Fresh leaves decoction is taken orally	Menstrual disorder
	Medic Brassicaceae			
14	*Nasturtium officinale* R.Br. Brassicaceae	Leaves	Fresh leaves are cooked as vegetables and taken orally	Constipation
15	*Bauhinia variegate* L. Caesalpiniodeae	Leaves, flowers	Leaves and flowers paste is give to cattle	Diarrhoea
16	*Silene conoidea* L. Caryophyllaceae	Leaves	Fresh leaves paste is applied topically	Skin infection
17	*Stellaria media* (L.). Cyr. Caryophyllaceae	Leaves	Fresh leaves paste is applied topically	Swelling joints, broken bones
			Fresh leaves decoction is taken orally	Constipation
18	*Chenopodium album* L. Chenopodiaceae	Leaves	Fresh leaves are cooked as vegetable and eaten raw.	Constipation, intestinal worms
			Fresh leaves are grinded and mixed with water and sugar. This juice is taken orally	Jaundice, urinary disorder
19	*Commelina benghalensis* L.	Roots	Dried roots are grinded and powder is taken orally	Epilepsy
	Commelinaceae		Fresh roots decoction is taken orally	Stomach disorders
20	*Evolvulus alsinoides* L.	Leaves	Leaves are crushed and mixed along with water. This juice is taken orally	Indigestion, constipation
	Convolvulaceae			
21	*Dioscorea deltoidea* Wall. ex, Kunth.	Tubers	Tubers are crushed and paste is taken orally and topically	Intestinal worms and lice
	Dioscoreaceae			
22	*Lamium amplexicaule* L.	Leaves	Fresh leaves paste is applied topically	Joints swelling
	Lamiaceae			
23	*Origanum vulgare* subsp. *Hirtum* L.	Leaves	Fresh leaves are chewed	Toothache and mouth gums
	Lamiaceae			
24	*Tulip stellata var*. *clusiana* Hk. f.,	Bulbs	Fresh bulbs are pealed off and taken orally	Heart problem
	Liliaceae			
25	*Malva parviflora* L.	Leaves	Fresh leaves decoction is taken orally	Constipation, cough, fever
	Malvaceae			
26	*Ficus carica* L.	Leaves	Fresh leaves are crushed and paste is applied topically	Boils
	Moracea	Latex	Fresh milky latex is applied topically	Watts
27	*Ficus palmata* Forssak.	Leaves	Fresh leaves are boiled in the milk of goat and taken orally	Bowel complaints
	Moracea	Latex	Milky latex is applied topically	Warts, small tumours
28	*Oxalis corniculata* L.	Leaves	Fresh leaves are crushed and paste is applied topically	Worms and scorpion sting
	Oxalidaceae			
29	*Lathyrus aphaca* L.	Seeds	Dried seeds powder is mixed in tobacco	Narcotic, soothing effect
	Papilionoideae			
30	*Medicago polymorpha* L.	Leaves	Fresh leaves are cooked in water and taken orally	Constipation, indigestion
	Papilionoideae			
31	*Melilotus albus* Medik.	Leaves	Fresh leaves paste is applied topically	Inflammation, joint pain
	Papilionoideae			
32	*Melilotus indicus* (L.)	Leaves	Fresh leaves paste is applied topically	Joint swelling
	All. Papilionoideae			
33	*Vicia faba* L.	Leaves	Fresh leaves decoction is taken orally	Kidney pain, eye infection
	Papilionoideae			
34	*Vicia sativa* L.	Leaves	Leaves paste is applied topically	Scorpion sting
	Papilionoideae			
35	*Plantago lanceolata* L.	Leaves	Fresh leaves paste is applied topically	Sores
	Plantaginaceaea	Seed husk	Seed husk along with sugar (Gur) is mixed in water and taken orally	Jaundice, internal body inflammation, constipation
36	*Polygonum amplexicaule* D. Don, Prodr.	Leaves	Fresh leaves are boiled in water along with sugar and decoction is taken orally	Fever, joint pain, flue
	Polygonaceae			
37	*Polygonum aviculare* L.	Leaves	Fresh leaves decoction is taken orally	Diarrhoea, dysentery
	Polygonaceae			
38	*Rumex dentatus* L.	Leaves	Fresh leaves are applied topically	Stinging nettle.
	Polygonaceae			
39	*Rumex hastatus* D. Don, Prodr.	Leaves and roots	Fresh leaves are roots are crushed and mixed in water. This extract is taken orally	Jaundice
	Polygonaceae			
40	*Portulaca quadrifida* L.	Leaves	Fresh leaves are slightly wormed and applied topically	Joint swelling
	Portulaceaea			
41	*Galium aparine* L.	Leaves	Fresh leaves paste is applied topically	Wounds healing
	Rubiaceae		Extract of fresh leaves is taken orally.	Jaundice
42	*Veronica arvensis* L.	Leaves	Fresh leaves decoction is taken orally	Skin infection, blood purifier
	Scrophulariaceae			
43	*Solanum nigrum* L.	Leaves	Leaves are crushed and mixed in water. This extract is applied topically	Washing painful eyes
	Solanaceae			
44	*Pimpinella diversifolia* (Wall.) DC. Prodor.	Leaves	Dried leaves are grinded along with salt and powder is taken orally	Gas trouble, indigestion
	Umbelliferae			
45	*Torilis leptophylla* (L.)	Leaves	Dried leaves powder is taken orally with water	Gastrointestinal disorders
	Reichb.f. Umbelliferae			

### Plant parts used and mode of consumption

Different parts of wild edible vegetables were consumed in diverse ways according to local traditions (Table 1). Wild edible vegetables are cooked as fresh in water (51%), e.g. *Amaranthus* spp. *Digera muricata*, *Bidens bipinnata*, *Capsella bursa*-*pastoris*, *Nasturtium officinale*, *Stellaria media*, *Commelina benghalensis*, *Malva parviflora*, *Lathyrus aphaca*, *Medicago polymorpha*, *Melilotus* spp. *Vicia* spp. *Portulaca quardifida*, *Galium aprine*, *Solanum nigrum* and *Torilis leptophyll*; in diluted milk (42%) e.g. *Cichorium intybus*, *Launaea procumbens*, *Sonchus* spp. *Taraxacum officinale*, *Bauhinia variegate*, *Silene conoidea*, *Tulip stellata*, *Plantago lanceoplata*, *Bistorta amplexicaulis*, *Pimpinella diversifolia*, *Ficus* and *Rumex* species; and in both water and diluted milk (Lussii) (7%) e.g. *Ficus* and *Sonchus* species. Flowers and rhizome of some vegetables species were also consumed fried in vegetable oil or ghee such as *Bombax malabaracum* and *Dioscorea deltoidea*. Present findings are in agreement to [19] regarding plants consumed cooked in several Mediterranean regions.

### Medicinal uses of wild edible vegetables species

No one knows when or where plants first began to be used in the treatment of diseases, but the grave of a Neanderthal man buried 60,000 years ago, revealed that connection between plants and health has existed for thousands of years [29]. The northern mountains of Pakistan are well known for their biodiversity as they are located at the intersection of great Karakorum, Himalaya and Hindu Kush ranges. These three mountain ranges together contain 25,000 species (about 10% of the world floras), out of which around 10,000 are economically or medicinally useful. Estimated total flora of Pakistan is comprised of 6000 species, out of which more than 4000 plant species grow in mountainous regions of Hindukush and Himalayas [30,31]. Over 75% of population in Pakistan is cured by means of traditional medicines, prescribed by more than 50,000 traditional herb practitioners and the traditional knowledge of plant based medications pass down from family to family of herbalists and within communities [32].

**Figure 2:**
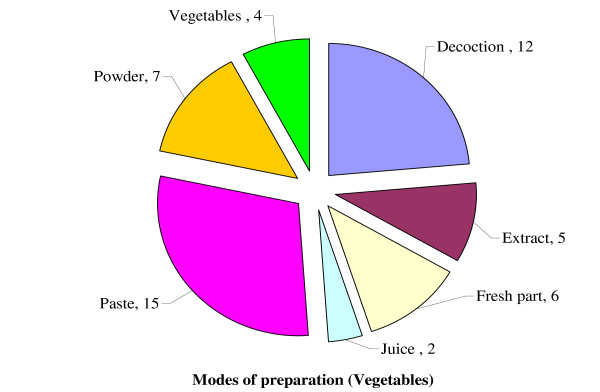
Modes of preparation.

During present survey decline was categorically observed in the trends of using conventional phytotherapies which is obviously because, the younger generation usually consider the belief in plant remedies a sort of superstition and less efficient compared to modern medicine. It was observed that leaves are highly utilized (70%) plant parts followed by seeds (10%), roots (6%), latex (4%), bark, bulb, flowers, tubers and rhizomes (2% each). Data presented in (Table 1), revealed that a total 51 recipes based on wild edible vegetables were used by the inhabitants of study sites. These medications can be divided into two categories: single plant based and from more than one plant based medications. In majority of the cases water is used as medium for preparation while milk, ghee, oil, egg and butter are used for application which is corroborated with [33]. Modes of preparation falls into different categories (Figure 2), such as paste of plant parts (29%) was common mode of recipes followed by decoction (24%), powder (14%), eaten fresh (12%), extract (10%), cooked vegetable (8%) and juice (4%). Mostly the mode of application falls in two categories topical as well as oral. Oral medications are taken along with water, milk or black tea. In regard to the patient condition, the preparations are applied more than two times each day until control.

**Figure 3:**
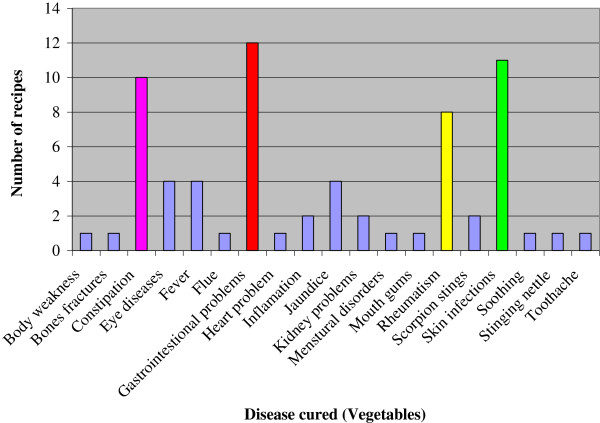
Number of recipes anddiseases cured by wild vegetables species.

The local inhabitants identified different types of ailments including gastrointestinal disorders (abdominal pain, gas trouble, gastric ulcer, intestinal worms, constipation, vomiting, diarrhea, dysentery), respiratory problems (asthma, flue, throat ache, cough), skin infections (measles, mouth gums, rashes, wound healing), bone fracture, rheumatism, diabetes, earache, tooth ache, eye infection, fever, heart problems, inflammation, jaundice, kidney problems, menstrual disorders, milk production, piles, scorpion sting and general weakness which were treated through different plant based remedies; twelve medications were used to cure gastrointestinal disorders followed by eleven to treat skin infections, ten against constipation, eight to cure rheumatism, four for each eye diseases, fever and jaundice, two for each inflammation, kidney problems and scorpion sting, where as other diseases were treated by one recipe each (Figure 3).

Medicinal uses of wild edible vegetables were compared with the available ethnobotanical literature which indicated that present applications of *Cichorium intybus*, *Stellaria media*, *Launaea procumbens*, *Chenopodium album*, *Dioscorea deltoidea*, *Oxalis corniculata*, *Lathyrus aphaca*, *Vicia sativa*, *Plantago lanceoplata*, *Rumex dentatus*, *Rumex hastatus*, *Solanum nigrum*, *Pimpinella diversifolia* and *Torilis leptophylla* were in agreement to [30,33-53]. Seed powder of *Amaranthus hybridus*, *A*. *spinosus* and *A*. *viridis* was used to improve eye vision problems, whereas according to [53,54] leaves of *Amaranthus hybridus* were used for internal inflammation, headache, stomach pain and to antidote snake and scorpion sting. Leaves of *Amaranthus viridis* and *A*. *spinosus* were found effective against scorpion sting, skin infections, mouth gums, piles, tooth ache diarrhoea and as laxative [43,47,52,55,56]. Extract from the leaves of *Bidens bipinnata* is used to cure leprosy and skin cuts, while same species was reported against stomach problems, menstruation, pain, influenza, scurvy, rheumatism, diarrhoea and for cold [44,53,57]. Leaf decoction of *Sonchus asper* and *S*. *oleraceous* are used against constipation and for body weakness, whereas [42,53] documented that leaves of *S*. *oleraceous* were effective against internal inflammation and wound healing. According to [44,45,48,55,58] leaves of *S*. *asper* were found useful for stomach problems and Jaundice.

Decoction of *Taraxacum officinale* rhizome is used to cure jaundice, whereas [41,52] mentioned that same species is used as aperients, diuretic, tonic, to cure constipation, kidney and liver disorders. Medicinal uses of *Bombax malabaracum* were found similar to that of reported by [30,33,57]. Local inhabitants of Lesser Himalayas use leaf decoction of *Capsella bursa*-*pastoris* to cure menstrual disorders, whereas [57] reported that same species was effective for heat cleaning. Leaves of *Nasturtium officinale* were found effective against constipation, but [53,55] mentioned that leaves and stem of same species were used to cure hepatic pain, pneumonia, indigestion, kidney pain and to purify blood. Paste form leaves and flowers of *Bauhinia variegata* was found useful to control diarrhoea, whereas [52] documented that bark of the same plant was used to treat skin diseases. The leaf paste of *Silene conoidea* was used against skin infections, but [38] mentioned that leaf paste of same species was used to cure pimples and backache.

Tribal communities of Himalayas use roots of *Commelina benghalensis* to cure epilepsy and stomach disorders, whereas [54] documented that leaves of the same species were found effective against liver complaints, snake and scorpion sting. Juice extracted from the leaves of *Evolvulus alsinoides* was found effectual against constipation and indigestion, whereas [47] recognized that leaves of this species were used against asthma and bronchitis. Leaves of *Origanum vulgare* were used to cure mouth gums and toothache, while according to [4,52] same species is used to alleviate stomach problems and sore throat. Leaf decoction of *Malva parviflora* was used to treat constipation, cough and fever, but according to [56] same plant is applied on swellings wounds and sores. Leaf paste and milky latex of *Ficus carica* and *F*. *palmata* were used to cure boils, warts, tumours and bowel complaints, whereas fruits of these species were found laxative [52,59]. Leaf decoction of *Polygonum amplexicaule* was found effective against fever, joint pain and flue; whereas [40] reported that leaves and shoots of same species were used for curing ulcer, sore throat, inflammation of mouth and tongue.

Fresh leaves of *Portulaca quadrifida* were applied topically on swelling joints, but according to [60] whole plant was used to cure asthma. Leaf paste and extract of *Galium aparine* were found effective for wound healing and jaundice, whereas [61] recognized that same species was used to cure injuries, skin infections, as tonic and diuretic. To our knowledge medicinal uses of *Digeramuricata*, *Dryopteris ramosa*, *Lamium amplexicaule*, *Tulipa stellata*, *Medicago polymorpha*, *Melilotus albus*, *Melilotus indicus*, *Vicia faba*, *Polygonum aviculare* and *Veronica arvensis* documented during present work were found hardly ever reported in adjacent areas and other parts of the world.

### Ethnobotanical uses of wild edible vegetables

**Figure 4:**
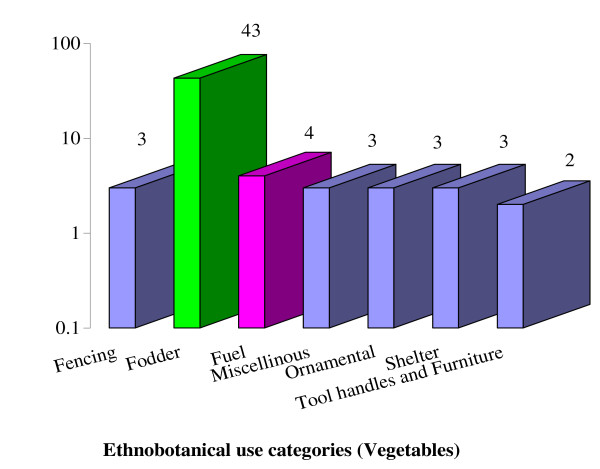
Ethnobotanical use categories and their number.

Among wild edible vegetables 43 species (95%) were used as fodder and forage for life stock, 4 species (9%) were used as fuel, 3 species each (7%) of wild vegetables such as *Ficus carica*, *F*. *palmata* and *Bauhinia variegata* were used by the local inhabitants of Lesser Himalayas in making shelters, in fencing and hedging, as ornamental plant and for miscellaneous purpose, 2 species (4%) were used in making tools handles and furniture (Figure 4). Ethnobotanical uses of wild edible vegetables reported during present investigation were found in agreement to [30,35,39,55].

### Species’ cultural importance

**Figure 5:**
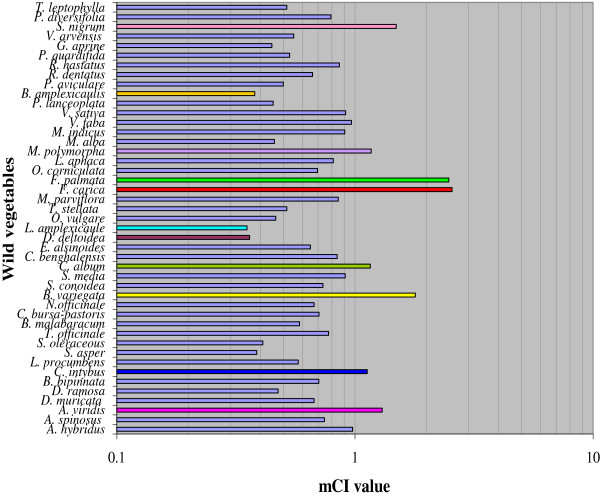
Mean cultural index of wild edible vegetables.

The terms “cultural importance” and “relative importance” usually are used interchangeably in the literature to refer to the importance of certain plants to a given culture [62]. The cultural importance index (CI) explains not only the spread of the uses (number of informants) for each species, but also it’s worth [63]. It can be assumed that the CI index is a proficient tool for highlighting those species with a high-agreement for the survey culture and so to recognize the shared knowledge of the peoples. During present study CI index and mean cultural importance index were computed to measure the cultural values of each wild edible vegetable in five studied sites [Additional file 1: Table S1], which can be used to evaluate the plant awareness between diverse cultures [11] and to study the intra cultural variations. On the bases of use reports (UR) the cultural importance index (CI) and mean cultural index (mCI) of wild edible vegetables within the five study localities (Margalla Hills, Haripur, Abbottabad, Murree and Mansehra) of Lesser Himalayas were intended. Among all wild edible vegetables *Ficus carica* was found most cited species followed by *Ficus palmata*, *Bauhinia variegata*, *Solanum nigrum*, *Amaranthus viridis*, *Medicago polymorpha*, *Chenopodium album*, *Cichorium intybus*, *Amaranthus hybridus* and *Vicia faba* (Figure 5). All These species are used as food, medicines, fodder, as fuel wood, in construction, sheltering, fencing and making agricultural tools. Some of these plants are considered as holy plants being mentioned in holy books (e.g., *Ficus carica* and *Ficus palmata* in Quraan). It was also observed that because of cultural values or decision, local inhabitants use only a small part of their natural flora present in their surrounding.

### Difference in cultural index (CI) values for species among the different sites

Results of cultural importance index and mean cultural importance index of 45 wild edible vegetables mentioned in descending order (Additional file 1: Table S1) confirmed substantial differences among the CI values obtained in the different areas. The top ten species of wild edible vegetables with the highest mCI, were cited in all the five surveyed areas and most were important in every site. The next thirty two species were also used at all studied regions, *Vicia sativa*, *Lamium amplexicaule*. These are based on a common cultural background. Interestingly CI values for wild edible vegetables species in Margalla Hills (50.30) and district Haripur (44.26) were found higher than for species in other areas (Additional file 1: Table S1). These findings exhibited that the traditional knowledge of wild edible plants and plant collection are much spread in isolated areas compare to urban sites [11]. To scrutinize this further we can calculate the mean of the CI values for all the species in each study site (mCIa) as a measure of botanical knowledge. It was found that mCIa value for Margalla Hills (1.117) was more than that of the values: 0.983 for Haripur, 0.774 for Mansehra, 0.693 for Abbottabad and 0.486 for Murree. This shows a great difference in the traditional knowledge of wild edible plants among different human groups. Although Mansehra, Abbottabad and Murree are adjoining and share similar environment, the difference is noteworthy and might be due to loss of knowledge in the former. Moreover high mCIa values in the isolated areas of Margalla Hills, Haripur and Mansehra indicates more dependence of inhabitants on surrounding natural flora.

### Cultural importance of the families

**Figure 6:**
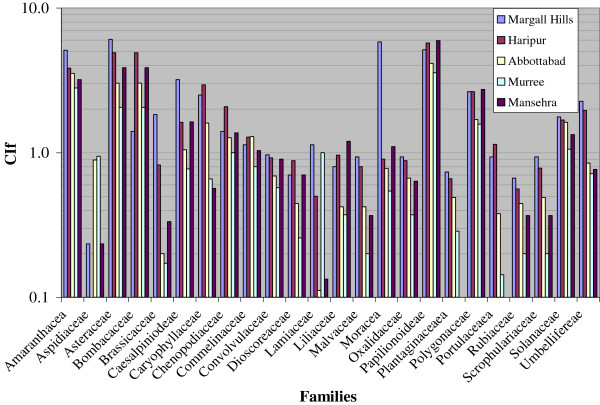
Cultural importance index of wild edible vegetables’ families (CIf).

A comparison between the cultural indexes of most quoted families (CIf) of wild edible vegetables mentioned in (Figure 6) revealed that Papilionoideae, with six species was found most quoted botanical family because members of this family were consumed as food, fodder and in medicines. Other remarkable families of wild vegetables within top ten were Asteraceae with six species, being consumed as vegetables and in medicines, followed by Amaranthaceae (4 species), Bombacaceae (1 species), Moraceae (2 species), Polygonaceae (4 species), Caesalpiniodeae (1 species), Caryophyllaceae (2 species), Solanaceae and Chenopodiaceae (1 species each). Remaining families of vegetables showed low representation and ethno-medicinal values. Present findings are in agreement with those of [19], who recognized that Asteraceae, Rosaceae and Umbelliferae were among the most significant families of wild edible plants in the Mediterranean regions. Hence present results confirm that local people tend to use preferably the plants that are accessible to them. These observations corroborated with those of [64-67].

### Socio-economic significance

Income derived from the sale of wild plant species is very important for poor households in order to meet basic needs [28]. In addition to food and therapeutic values, some of the studied species are also marketable and provide the chance of additional household income. During present survey it was observed that 17.7% of wild edible vegetables e.g., *Dryopteris ramosa*, *Nasturtium officinale*, *Bauhinia variegata*, *Chenopodium album*, *Malva parviflora*, *Portulaca quardifida* and *Solanum nigrum* were soled as vegetables at local markets of Abbottabad, Rawalpindi and Haripur (Additional file 2: Table S2), 0.04% e.g., *Ficus carica*, *Ficus palmata* are marketed as edible fruits and 0.06% e.g., *Bauhinia variegata*, *Ficus carica* and *Ficus palmata* are soled as fuel wood locally.

### Threats to wild edible plants

Present investigation revealed that mostly wild edible vegetables are collected from waste lands (44.5%), agricultural fields (33.4%), forests (20%) and water bodies (2.2%). These plant species were found under threats in their natural habitats because of different human activities. According to local inhabitants agricultural land expansion, over-harvesting, over-grazing, uncontrolled fire setting and fuel wood collection are among the common threats to these alternative food resources and their impacts varies from place to place. In order to find out local inhabitants perception on threats to wild edible vegetables, pair wise ranking of five factors (agricultural land expansion, over-harvesting, over-grazing, uncontrolled fire setting and fuel wood collection) were conducted.

However, the overall rating for all communities mentioned in Table 2, demonstrated agricultural land expansion as the dominating threat to wild edible vegetables, followed by over-harvesting, over-grazing, fire and fuel wood collection. Majority of the wild edible vegetables have no protection except, *Bauhinia variegata*, *Chenopodium album*, *Solanum nigrum*, *Ficus carica* and *Ficus palmata* which are protected, cultivated and marketed by some formers. This shows that achievement of economic payback from plant species might endorse local people’s interest in the conservation, maintenance and preservation of significant and endangered species [28].

**Table 2:** Pair wise ranking of factors considered as threats to wild edible vegetables

**Factors**	**Respondents**																				**Total**	**Rank**
	MH^*^	**MH**	**MH**	**MH**	H^*^	**DH**	**DH**	**DH**	AB^*^	**AB**	**AB**	**AB**	Mu^*^	**Mu**	**Mu**	**Mu**	Mn^*^	**Mn**	**Mn**	**Mn**		
	**1**	**2**	**3**	**4**	**1**	**2**	**3**	**4**	**1**	**2**	**3**	**4**	**1**	**2**	**3**	**4**	**1**	**2**	**3**	**4**		
Agricultural land expansion	4	3	2	3	6	5	3	7	3	4	2	1	3	2	3	3	4	2	3	2	65	1
Over harvesting	2	3	2	3	3	4	2	4	2	1	3	2	1	3	1	4	3	1	2	4	50	2
Over grazing	1	3	1	2	2	1	3	1	1	2	1	3	2	1	3	1	2	3	1	2	36	3
Fire	3	2	4	1	3	2	1	2	1	1	0	1	2	1	1	2	1	0	2	1	31	4
Fuel	2	1	0	2	1	2	0	1	3	1	1	0	1	2	1	0	0	1	1	0	20	5

^*^MH Margalla Hills National Park, ^*^H Haripur, ^*^AB Abbottabad,^*^Mu Murree, ^*^Mn Mansehra. The score mentioned in the table is based on the information from 16 key informants at four sub sites from each study area.

## Conclusion

Major populace of Lesser Himalayas still use wild edible plants as food and to cure various ailments but, this traditional knowledge have severely eroded due to change in the life style of local inhabitants, which needs to be documented before it is too late. Present investigation revealed that patterns of wild edible plant usage depend mainly on socio-economic factors compare to climatic conditions or wealth of flora. Analysis of results indicated that in all the surveyed areas, most of the plants are consumed by poor families during normal and difficult times. However, decline in use of some species may lead to the diminishing of the traditional knowledge about such plants. Use reports and citation of majority of wild edible vegetables, confirmed a universal cultural heritage in the study sites regarding the gathered food plants, because most of the quoted taxa are same and their cultural worth is analogous. However, a few differences in the cultural importance indices of wild edible vegetables were also observed, which may be due to the similar live hood and difference in indigenous knowledge of the local communities. *Ficus carica*, *Ficus palmata*, *Bauhinia variegata* and *Solanum nigrum* exhibited maximum cultural importance index (CI). Present findings also revealed that many wild edible vegetables species are under pressure from various anthropogenic factors, demand public awareness, community based management and urgent collection of germplasm. Further exploration is suggested into nutritional profile, phytochemical analysis, antioxidant potential, essential and toxic components in conventional food resources; pharmacological applications; dietary requirements; skill training in farming and biotechnological techniques to improve yields.

## Competing interests

The authors declare that they have no competing interests.

## Authors’ contribution

a) AMA: Present paper is a part of my PhD work, I have Carried out field survey, sampling, data collection and compilation of manuscript. b) MAK: Supervised the work. c) MHS: Supervised the work and technical checking the manuscript. d) MMS: Help in field work and Moral Support. e) AP: Support in data collection. f) MA: Plants identification. All authors read and approved the final manuscript, there is no objection or further suggestion.

## Supplementary Material

Additional file 1: Table S1 Cultural important index (CI), mean cultural importance index (mCI) of wild edible vegetables. Click here for file

Additional file 2: Table S2 Socio-economic values of wild edible vegetables. Click here for file
