# Supplementary material for: Ethnobotanical appraisal and cultural values of medicinally important wild edible vegetables of Lesser Himalayas-Pakistan
Source: J Ethnobiol Ethnomed. 2013 Sep 14;9:66. doi: 10.1186/1746-4269-9-66 (PMC3853161; doi:10.1186/1746-4269-9-66)
Supplement: Additional file 1: Table S1 — Cultural important index (CI), mean cultural importance index (mCI) of wild edible vegetables. [file 1746-4269-9-66-S1.doc]

Additional file 1: Table S1: Cultural important index (CI), mean cultural importance index (mCI) of wild edible vegetables

| S. No | Botanical name | Margalla Hills | Haripur | Abbottabad | Murree | Mansehra | mCI |
| --- | --- | --- | --- | --- | --- | --- | --- |
|  | *Ficus carica* | 2.967 | 2.900 | 2.067 | 1.829 | 3.033 | 2.559 |
|  | *Ficus palmata* | 2.867 | 2.820 | 2.067 | 1.743 | 2.900 | 2.479 |
|  | *Bauhinia variegata* | 3.200 | 2.940 | 1.600 | 0.657 | 0.567 | 1.793 |
|  | *Solanum nigrum* | 1.767 | 1.680 | 1.622 | 1.057 | 1.333 | 1.492 |
|  | *Amaranthus viridus* | 1.933 | 1.120 | 1.089 | 1.229 | 1.133 | 1.301 |
|  | *Medicago polymorpha* | 1.367 | 1.300 | 1.156 | 0.829 | 1.200 | 1.170 |
|  | *Chenopodium album* | 1.400 | 1.280 | 1.289 | 0.800 | 1.033 | 1.160 |
|  | *Cichorium intybus* | 1.433 | 1.140 | 0.956 | 0.943 | 1.167 | 1.128 |
|  | *Amaranthus hybridus* | 1.133 | 1.180 | 1.044 | 0.657 | 0.867 | 0.976 |
|  | *Vicia faba* | 1.267 | 1.180 | 0.956 | 0.429 | 1.000 | 0.966 |
|  | *Vicia sativa* | 1.233 | 1.140 | 0.778 | 0.514 | 0.900 | 0.913 |
|  | *Stellaria media* | 1.233 | 1.180 | 0.800 | 0.600 | 0.733 | 0.909 |
|  | *Melilotus indicus* | 1.267 | 1.060 | 0.844 | 0.457 | 0.900 | 0.906 |
|  | *Rumex hastatus* | 1.067 | 1.200 | 0.622 | 0.714 | 0.700 | 0.861 |
|  | *Malva parviflora* | 0.933 | 0.900 | 0.778 | 0.543 | 1.100 | 0.851 |
|  | *Commelina benghalensis* | 1.133 | 0.920 | 0.689 | 0.571 | 0.900 | 0.843 |
|  | *Lathyrus aphaca* | 1.067 | 0.840 | 0.689 | 0.429 | 1.033 | 0.811 |
|  | *Pimpinella diversifolia* | 1.433 | 1.240 | 0.511 | 0.514 | 0.267 | 0.793 |
|  | *Taraxacum officinale* | 0.967 | 0.900 | 0.778 | 0.400 | 0.833 | 0.776 |
|  | *Amaranthus spinosus* | 0.767 | 0.760 | 1.089 | 0.371 | 0.733 | 0.744 |
|  | *Silene conoidea* | 1.267 | 0.900 | 0.467 | 0.400 | 0.633 | 0.733 |
|  | *Capsella bursa-pastoris* | 0.967 | 0.920 | 0.489 | 0.257 | 0.900 | 0.707 |
|  | *Bidens bipinnata* | 1.400 | 1.040 | 0.378 | 0.171 | 0.533 | 0.705 |
|  | *Oxalis corniculata* | 0.933 | 0.880 | 0.667 | 0.371 | 0.633 | 0.697 |
|  | *Nasturtium officinale* | 0.867 | 0.700 | 0.556 | 0.514 | 0.733 | 0.674 |
|  | *Digeria muricata* | 1.267 | 0.780 | 0.311 | 0.543 | 0.467 | 0.673 |
|  | *Rumex dentatus* | 0.700 | 0.700 | 0.644 | 0.743 | 0.533 | 0.664 |
|  | *Evolvulus alsinoides* | 0.967 | 0.880 | 0.444 | 0.257 | 0.700 | 0.650 |
|  | *Bombax malabaracum* | 1.400 | 0.820 | 0.200 | 0.171 | 0.333 | 0.585 |
|  | *Launaea procumbens* | 0.967 | 0.800 | 0.422 | 0.200 | 0.500 | 0.578 |
|  | *Veronica arvensis* | 0.933 | 0.780 | 0.489 | 0.200 | 0.367 | 0.554 |
|  | *Portulaca quardifida* | 0.933 | 1.140 | 0.378 | 0.143 | 0.067 | 0.532 |
|  | *Tulip stellata* | 0.800 | 0.800 | 0.422 | 0.200 | 0.367 | 0.518 |
|  | *Torilis leptophylla* | 0.833 | 0.720 | 0.333 | 0.200 | 0.500 | 0.517 |
|  | *Polygonum aviculare* | 0.867 | 0.740 | 0.378 | 0.086 | 0.433 | 0.501 |
|  | *Dyropteris ramosa* | 0.233 | 0.080 | 0.889 | 0.943 | 0.233 | 0.476 |
|  | *Origanum vulgare* | 0.500 | 0.500 | 0.156 | 0.171 | 1.000 | 0.465 |
|  | *Plantago lanceoplata* | 0.733 | 0.660 | 0.489 | 0.286 | 0.100 | 0.454 |
|  | *Galium aprine* | 0.667 | 0.560 | 0.444 | 0.200 | 0.367 | 0.448 |
|  | *Sonchus oleraceous* | 0.633 | 0.500 | 0.267 | 0.286 | 0.367 | 0.410 |
|  | *Sonchus asper* | 0.667 | 0.520 | 0.222 | 0.057 | 0.467 | 0.387 |
|  | *Lamium amplexicaule* | 0.633 | 0.460 | 0.267 | 0.200 | 0.200 | 0.352 |
|  | *Dioscorea deltoidea* | 0.700 | 0.500 | 0.111 | 0.000 | 0.133 | 0.289 |
|  | *Melilotus alba* | 0.000 | 0.200 | 0.311 | 0.000 | 0.867 | 0.276 |
|  | *Bistorta amplexicaulis* | 0.000 | 0.000 | 0.044 | 0.029 | 1.067 | 0.228 |
|  | *Sum total* | 50.30 | 44.26 | 31.20 | 21.91 | 34.83 |  |
