# Supplementary material for: Ethnobotanical appraisal and cultural values of medicinally important wild edible vegetables of Lesser Himalayas-Pakistan
Source: J Ethnobiol Ethnomed. 2013 Sep 14;9:66. doi: 10.1186/1746-4269-9-66 (PMC3853161; doi:10.1186/1746-4269-9-66)
Supplement: Additional file 2: Table S2 — Socio-economic values of wild edible vegetables. [file 1746-4269-9-66-S2.doc]

Additional file 2: Table S2: Socio-economic values of wild edible vegetables

| S.No | Botanical name | Fod | Med | Fdd | Ful | Sh | F | Fen | Orn | Mk |
| --- | --- | --- | --- | --- | --- | --- | --- | --- | --- | --- |
| 1 | *Amaranthus hybridus* | + | + | + | - | - | - | - | - | - |
| 2 | *Amaranthus spinosus* | + | + | + | - | - | - | - | - | - |
| 3 | *Amaranthus viridus* | + | + | + | - | - | - | - | - | - |
| 4 | *Digeria muricata* | + | + | + | - | - | - | - | - | - |
| 5 | *Dryopteris ramosa* | + | + | - | - | - | - | - | - | + |
| 6 | *Bidens bipinnata* | + | + | + | - | - | - | - | - | - |
| 7 | *Cichorium intybus* | + | + | + | - | - | - | - | - | + |
| 8 | *Launaea procumbens* | + | + | + | - | - | - | - | - | - |
| 9 | *Sonchus asper* | + | + | + | - | - | - | - | - | - |
| 10 | *Sonchus oleraceous* | + | + | + | - | - | - | - | - | - |
| 11 | *Taraxacum officinale* | + | + | + | - | - | - | - | - | - |
| 12 | *Bombax malabaracum* | + | + | - | + | - | - | + | + | - |
| 13 | *Capsella bursa-pastoris* | + | + | + | - | - | - | - | - | - |
| 14 | *Nasturtium officinale* | + | + | - | - | - | - | - | - | + |
| 15 | *Bauhinia variegata* | + | + | + | + | + | - | - | + | + |
| 16 | *Silene conoidea* | + | + | + | - | - | - | - | - | - |
| 17 | *Stellaria media* | + | + | + | - | - | - | - | - | - |
| 18 | *Chenopodium album* | + | + | + | - | - | - | - | - | + |
| 19 | *Commelina benghalensis* | + | + | + | - | - | - | - | - | - |
| 20 | *Evolvulus alsinoides* | + | + | + | - | - | - | - | - | - |
| 21 | *Dioscorea deltoidea* | + | + | + | - | - | - | - | - | - |
| 22 | *Lamium amplexicaule* | + | + | + | - | - | - | - | - | - |
| 23 | *Origanum vulgare* | + | + | + | - | - | - | - | - | - |
| 24 | *Tulip stellata* | + | + | + | - | - | - | - | - | - |
| 25 | *Malva parviflora* | + | + | + | - | - | - | - | - | + |
| 26 | *Ficus carica* | + | + | + | + | + | + | + | + | + |
| 27 | *Ficus palmata* | + | + | + | + | + | + | + | + | + |
| 28 | *Oxalis corniculata* | + | + | + | - | - | - | - | - | - |
| 29 | *Lathyrus aphaca* | + | + | + | - | - | - | - | - | - |
| 30 | *Medicago polymorpha* | + | + | + | - | - | - | - | - | - |
| 31 | *Melilotus alba* | + | + | + | - | - | - | - | - | - |
| 32 | *Melilotus indicus* | + | + | + | - | - | - | - | - | - |
| 33 | *Vicia faba* | + | + | + | - | - | - | - | - | - |
| 34 | *Vicia sativa* | + | + | + | - | - | - | - | - | - |
| 35 | *Plantago lanceoplata* | + | + | + | - | - | - | - | - | - |
| 36 | *Bistorta amplexicaulis* | + | + | + | - | - | - | - | - | - |
| 37 | *Polygonum aviculare* | + | + | + | - | - | - | - | - | - |
| 38 | *Rumex dentatus* | + | + | + | - | - | - | - | - | - |
| 39 | *Rumex hastatus* | + | + | + | - | - | - | - | - | - |
| 40 | *Portulaca quardifida* | + | + | + | - | - | - | - | - | + |
| 41 | *Galium aprine* | + | + | + | - | - | - | - | - | - |
| 42 | *Veronica arvensis* | + | + | + | - | - | - | - | - | - |
| 43 | *Solanum nigrum* | + | + | + | - | - | - | - | - | + |
| 44 | *Pimpinella diversifolia* | + | + | + | - | - | - | - | - | - |
| 45 | *Torilis leptophylla* | + | + | + | - | - | - | - | - | - |

Fod = Food, Med = Medicine, Ful = Fuel, Sh = Shelter, F = Furniture, Fen = Fencing, Orn = Ornamental, Mk = Marketing
